# Prenatal androgen exposure causes a sexually dimorphic transgenerational increase in offspring susceptibility to anxiety disorders

**DOI:** 10.1038/s41398-020-01183-9

**Published:** 2021-01-13

**Authors:** Sanjiv Risal, Maria Manti, Haojiang Lu, Romina Fornes, Henrik Larsson, Anna Benrick, Qiaolin Deng, Carolyn E. Cesta, Mina A. Rosenqvist, Elisabet Stener-Victorin

**Affiliations:** 1 Department of Physiology and Pharmacology, Karolinska Institutet, Stockholm, Sweden; 2 Department of Medical Epidemiology and Biostatistics, Karolinska Institutet, Stockholm, Sweden; 3 School of Medical Sciences, Örebro University, Örebro, Sweden; 4 Department of Physiology, Sahlgrenska Academy, University of Gothenburg, Gothenburg, Sweden; 5 School of Health Sciences, University of Skövde, Skövde, Sweden; 6 Department of Medicine, Solna, Centre for Pharmacoepidemiology, Karolinska Institutet, Stockholm, Sweden

**Keywords:** Physiology, Depression

## Abstract

If and how obesity and elevated androgens in women with polycystic ovary syndrome (PCOS) affect their offspring’s psychiatric health is unclear. Using data from Swedish population health registers, we showed that daughters of mothers with PCOS have a 78% increased risk of being diagnosed with anxiety disorders. We next generated a PCOS-like mouse (F_0_) model induced by androgen exposure during late gestation, with or without diet-induced maternal obesity, and showed that the first generation (F_1_) female offspring develop anxiety-like behavior, which is transgenerationally transmitted through the female germline into the third generation of female offspring (F_3_) in the androgenized lineage. In contrast, following the male germline, F_3_ male offspring (mF_3_) displayed anxiety-like behavior in the androgenized and the obese lineages. Using a targeted approach to search for molecular targets within the amygdala, we identified five differentially expressed genes involved in anxiety-like behavior in F_3_ females in the androgenized lineage and eight genes in the obese lineage. In mF_3_ male offspring, three genes were dysregulated in the obese lineage but none in the androgenized lineage. Finally, we performed in vitro fertilization (IVF) using a PCOS mouse model of continuous androgen exposure. We showed that the IVF generated F_1_ and F_2_ offspring in the female germline did not develop anxiety-like behavior, while the F_2_ male offspring (mF_2_) in the male germline did. Our findings provide evidence that elevated maternal androgens in PCOS and maternal obesity may underlie the risk of a transgenerational transmission of anxiety disorders in children of women with PCOS.

## Introduction

Polycystic ovary syndrome (PCOS) is recognized as a heterogeneous disorder affecting over 15% of women in the general population and over 25% of women with obesity^1,2^. Over 60% of women with PCOS are diagnosed with at least one psychiatric disorder^3,4^. Elevated circulating androgens is the most prominent feature of PCOS, which persists throughout reproductive life and even after menopause^5,6^, and reproductive, metabolic and psychiatric dysfunction are all positively correlated with hyperandrogenaemia^4–7^.

Prenatal androgen exposure has been proposed to have a potential causal influence on the development of neuropsychiatric disorders in children born to women with PCOS in a Swedish register-based studies^8–10^. Specifically, an increase in the risk of attention-deficit/hyperactive disorder (ADHD) and autism spectrum disorder (ASD) in daughters, and to a lesser extent in sons of women with PCOS has been found even when accounting for genetic factors^8^. Similarly, a recent study has reported that children born to women with PCOS had higher risk for childhood anxiety diagnoses^11^, and maternal PCOS, independently, and jointly with maternal obesity, has been shown to be associated with increased risks for almost all groups of psychiatric and mild neurodevelopmental disorders in offspring^12^. Whether the risk is different for daughters and sons remains to be investigated.

Prenatal androgenized rodent studies have demonstrated that maternal androgen excess may underpin the risk of developing anxiety disorders in female offspring and to a lesser extent in male offspring^13,14^, whereas diet-induced maternal obesity increases anxiety-like behavior only in the male offspring^14^. Using the prenatal androgenized mouse model combined with a diet-induced maternal obesity model we recently demonstrated that elevated maternal androgens, and to lesser extent maternal obesity, reprogram the fetus and induce a transgenerational increase in female offspring susceptibility to develop a PCOS-like phenotype^15^. To ascertain transgenerational inheritance, the phenotypic changes must be manifested in F_3_ offspring because F_1_ fetuses and the already developing germline (that will give rise to the F_2_) are also directly exposed^15^. Thus, whether elevated maternal androgens potentiate transgenerational susceptibility to anxiety-like behavior in adult female and male offspring has not been explored.

## Results

### Daughters of women with PCOS are diagnosed with anxiety disorders

**Fig. 1:**
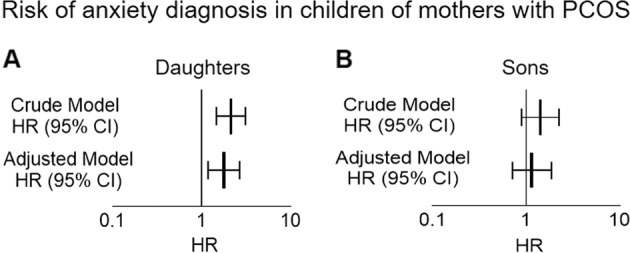
Risk of having an anxiety in daughters of women with PCOS. **A**, **B** Crude and adjusted hazard ratios (HRs) and 95% confidence intervals (CIs) for the association between maternal PCOS and daughter’s diagnosis of anxiety in the Swedish nation-wide register-based study. Adjusted model is adjusted for maternal age, maternal country of birth, maternal education, maternal and paternal psychiatric history, and year of birth of child.

We conducted a Swedish nationwide register-based cohort study to assess if daughters of women with PCOS have a higher risk of being diagnosed with anxiety disorders. By linking the Swedish Medical Birth Register (MBR) and the National Patient Register (NPR), a total of 102,466 children were identified, of which 8864 (8.7%) were born to mothers with a diagnosis of PCOS. Maternal and child characteristics are reported in Table S1. Overall, 1.32% of children (*n* = 117) born to a mother with PCOS had a diagnosis of anxiety made in inpatient or outpatient hospital-based specialized care, compared to 0.97% (*n* = 912) of children born to mothers without a PCOS diagnosis. The mean age (and standard deviation) of first anxiety diagnosis for all children was 13.5 ± 3.0 years, and 12.4 ± 3.1 years and 14.1 ± 2.8 years for boys and girls, respectively. Table S2 shows the crude and adjusted hazard ratios (HR). Children born to mothers with PCOS had an increased risk of being diagnosed with anxiety (adjusted model, HR = 1.49 95% CI 1.16–1.92). When adjusting the models for parental anxiety diagnoses, instead of any psychiatric diagnosis, the estimates remained similar (data not shown). When stratified by child’s sex (Table S2), daughters born to women with PCOS (*n* = 4290) had a 78% increased risk of being diagnosed with anxiety compared to daughters born to women without PCOS (adjusted model, HR = 1.78 95% CI 1.19–2.67). In contrast, sons born to women with PCOS (*n* = 4574) had no increase in risk for a diagnosis of anxiety (Adjusted HR = 1.15 95% CI 0.71–1.86) (Fig. 1a). The sensitivity analysis in the subset of the population with BMI information produced a similar pattern of estimates as the main analysis however the estimates were attenuated with wider confidence intervals (Table S3).

### Prenatal androgen exposure and maternal obesity cause transgenerational anxiety-like behavior

**Fig. 2:**
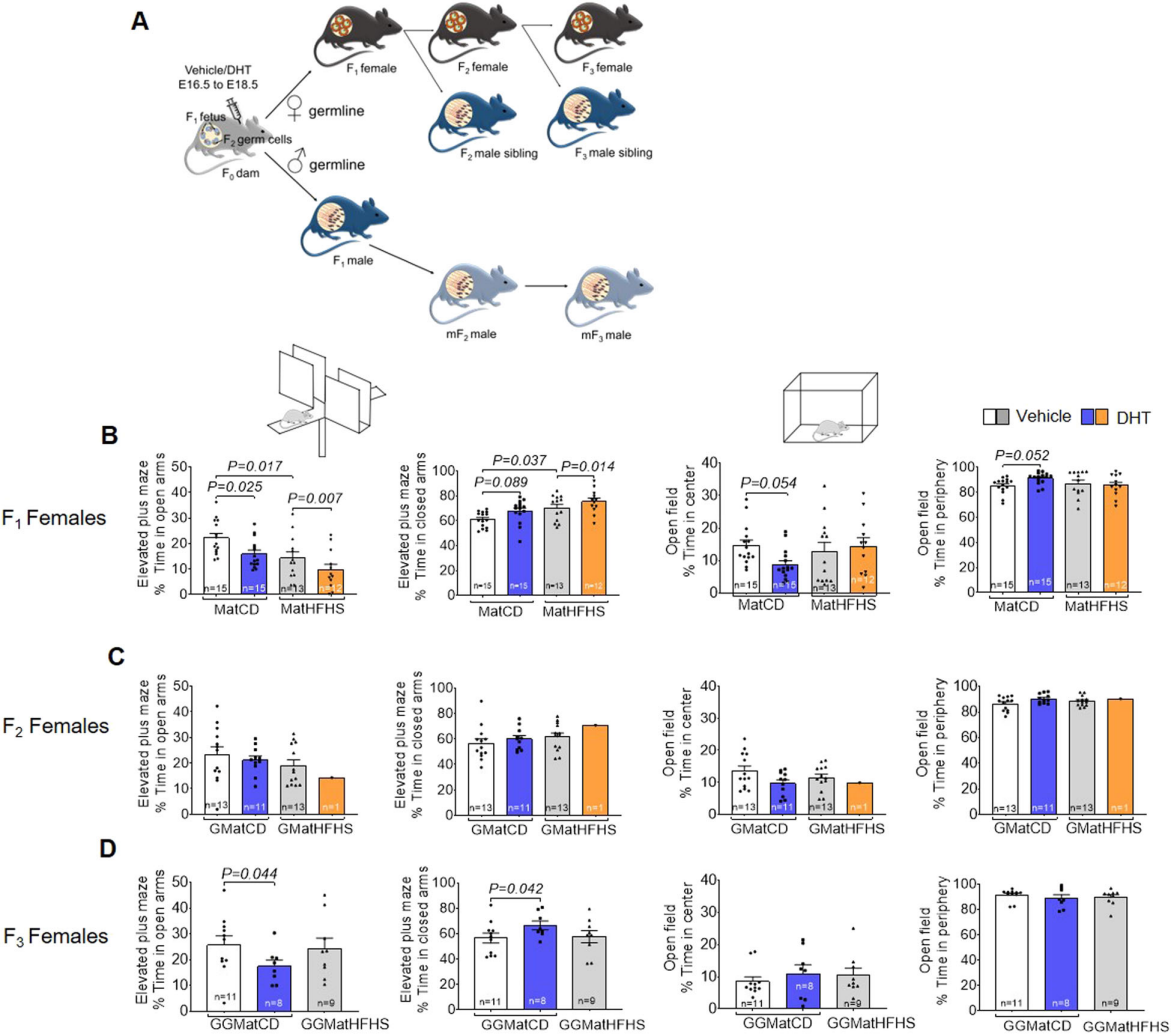
Prenatal androgen exposure causes transgenerational anxiety-like behavior in female offspring. **A** Schematic illustration of experimental design. Prior to mating with male mice fed control diet, F_0_ mothers were fed control diet or high fat-high sucrose (HFHS) diet for 6 weeks. During embryonic day (E) 16.5−E18.5 pregnant mice were injected subcutaneously with 250 µg of dihydrotestosterone (DHT), a non-aromatizable androgen, dissolved in 50 µL of sesame oil or 50 µL of sesame oil alone (vehicle) resulting in four lineages; CD+Vehicle (control); CD+DHT (androgenized); HFHS+Vehicle (obese); and HFHS+DHT (obese- androgenized). F_1_ female and male offspring were mated with unrelated males and females fed CD, respectively, to generate F_2_ and thereafter to generate F_3_ offspring. Anxiety-like behavior was tested in female and male offspring according to the graph. Male germline refers to F_2_ and F_3_ male offspring from F_1_ males. F_2_ and F_3_ male siblings are brothers to F_2_ and F_3_ female offspring. **B**–**D** Time spent in the open arms in the elevated plus maze (EPM); time spent in the closed arm of the EPM; time spent in the center of the open filed (OF); time spent in the periphery of the OF. F1: two-way ANOVA, Tukey’s post hoc analysis; F_2_ and F_3_: one-way ANOVA, Dunnett’s post hoc analysis. All data are presented as mean ± s.e.m. DHT dihydrotestosterone, CD control diet, HFHS high-fat high-sugar, Mat maternal, GMat grand-maternal, GGMat great-grand maternal. Numbers of mice are stated in the bars of each group.

**Fig. 3:**
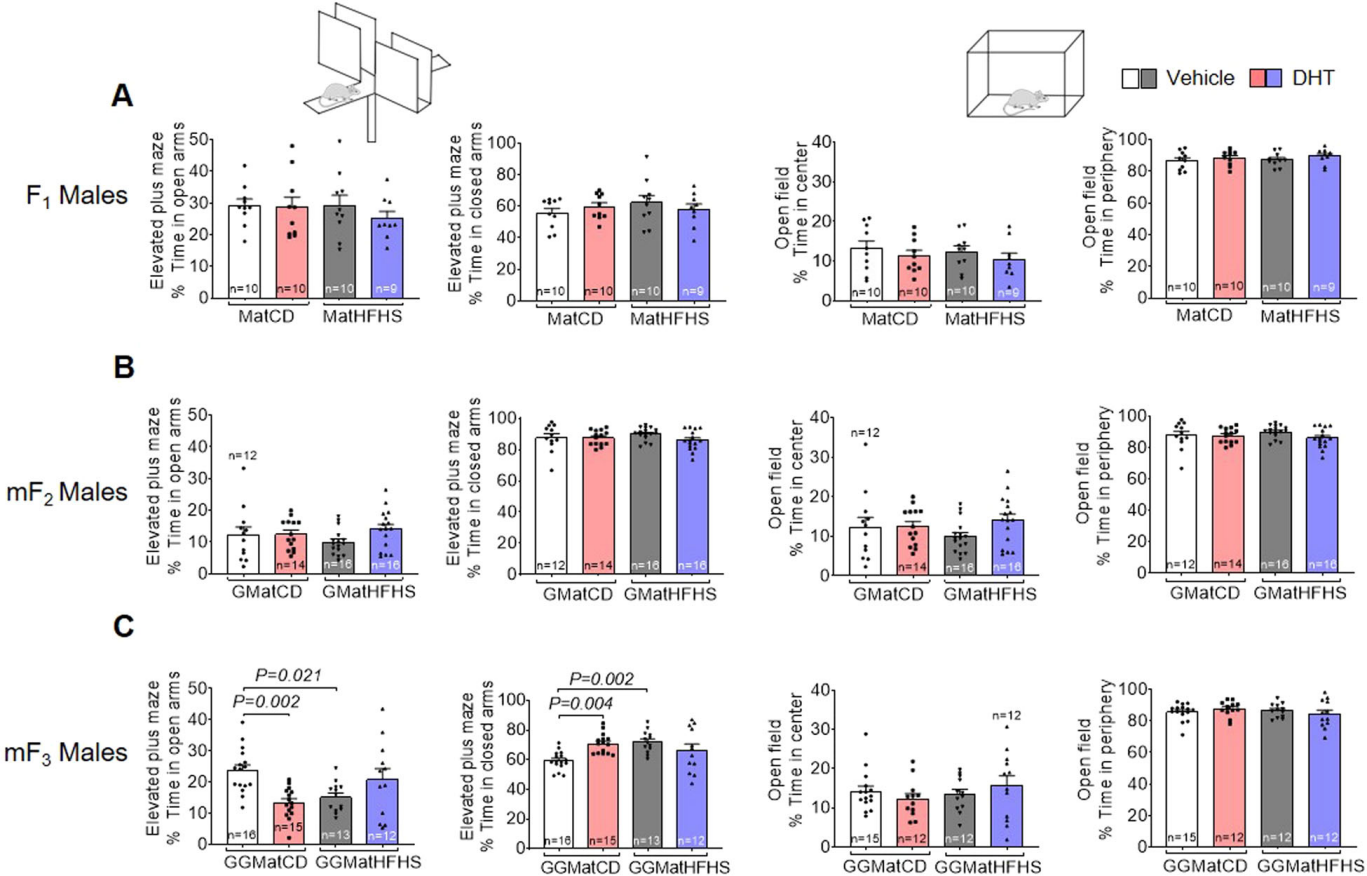
Prenatal androgen exposure with and without maternal obesity causes transgenerational anxiety-like behavior in male offspring following the male germline. **A**–**C** Time spent in the open arms in the elevated plus maze; time spent in the closed arms of the elevated plus maze; time spent in the center of the open field; time spent in the periphery of the open field. F_1_– mF_3_: two-way ANOVA, Tukey’s post hoc analysis. All data are presented as mean ± s.e.m. DHT dihydrotestosterone; CD control diet, HFHS high-fat high-sugar, Mat maternal, GMat grand-maternal, GGMat great-grand maternal. The numbers of mice are stated in the bars of each group.

Next, we aimed to elucidate whether female and male offspring (F_1_) of maternal androgen exposed mothers (F_0_) fed either control diet (CD) or high-fat high sugar diet (HFHS) are susceptible to develop anxiety-like behavioral traits in adulthood, and if the anxiety-like behavior phenotype is transmitted to the second (F_2_) and third-generation (F_3_) i.e., transgenerational (Fig. 2A and Table S4). F_1_ female offspring in the androgenized, the obese, and in the combined obese and androgenized lineages spent less time in the open arms and more time in the closed arms of the elevated plus maze (Fig. 2B), indicating that prenatal androgen exposure combined with maternal obesity aggravates anxiety-like behavior. There was a main effect of the androgenized lineage and the obese lineage in the elevated plus maze [open arms, androgenized: *F*_1,78_ = 11.5, *P* = 0.001 and obese: *F*_1,78_ = 19.28, *P* < 0.0001], [closed arms, androgenized: *F*_1, 78_ = 4.83, *P* = 0.006 and obese: *F*_1,78_ = 16.27, *P* = 0.0001]. Further, there was an interaction between the androgenized and the obese lineages in the open field, wherein the F_1_ female offspring in the androgenized lineage spent less time in the center [center: *F*_1,78_ = 4.86, *P* = 0.030] and more time in the periphery [periphery: *F*_1,78_ = 4.82, *P* = 0.031], while the F_1_ females in the combined lineages spent more time in the center and less time in the periphery of the open field (Fig. 2B). Furthermore, in line with human data, F_1_ male offspring did not present any anxiety-like behavioral changes (Fig. 3A).

Exploring the transgenerational transmission of anxiety-like behavior, we show that F_3_ female offspring in the androgenized lineage spent less time in the open arms and more time in the closed arms of the elevated plus maze, with no differences in the open field (Fig. 2D). No changes were observed in the F_2_ female offspring (Fig. 2C) and in the male F_2_ and F_3_ offspring, i.e., siblings (Supplementary Fig. 1A, B). The male germline, mF_3_ male offspring in the androgenized lineage and the mF_3_ male offspring in the obese lineage spent less time in the open arms, and more time in the closed arms of the elevated plus maze (Fig. 3C) but no difference in time spent in center and periphery of the open field. There was an interaction between the androgenized and the obese lineages in the elevated plus maze in mF_3_ male offspring [open arms: *F*_1,52_ = 14.93, *P* = 0.0003] and [closed arms: *F*_1,52_ = 12.35, *P* = 0.0009]. No changes were observed in the mF_2_ male offspring (Fig. 3B). These findings suggest that the anxiety-like behavioral phenotype can be transmitted across generations in female offspring from androgenized F_0_ mothers, but not in the male siblings. Further, mF_3_ male offspring following the male germline displays an anxiety-like behavior either due to maternal obesity or androgen exposure in F_0_ mothers.

### Prenatal androgen exposure and obesity resulted in altered amygdala gene expression

**Fig. 4:**
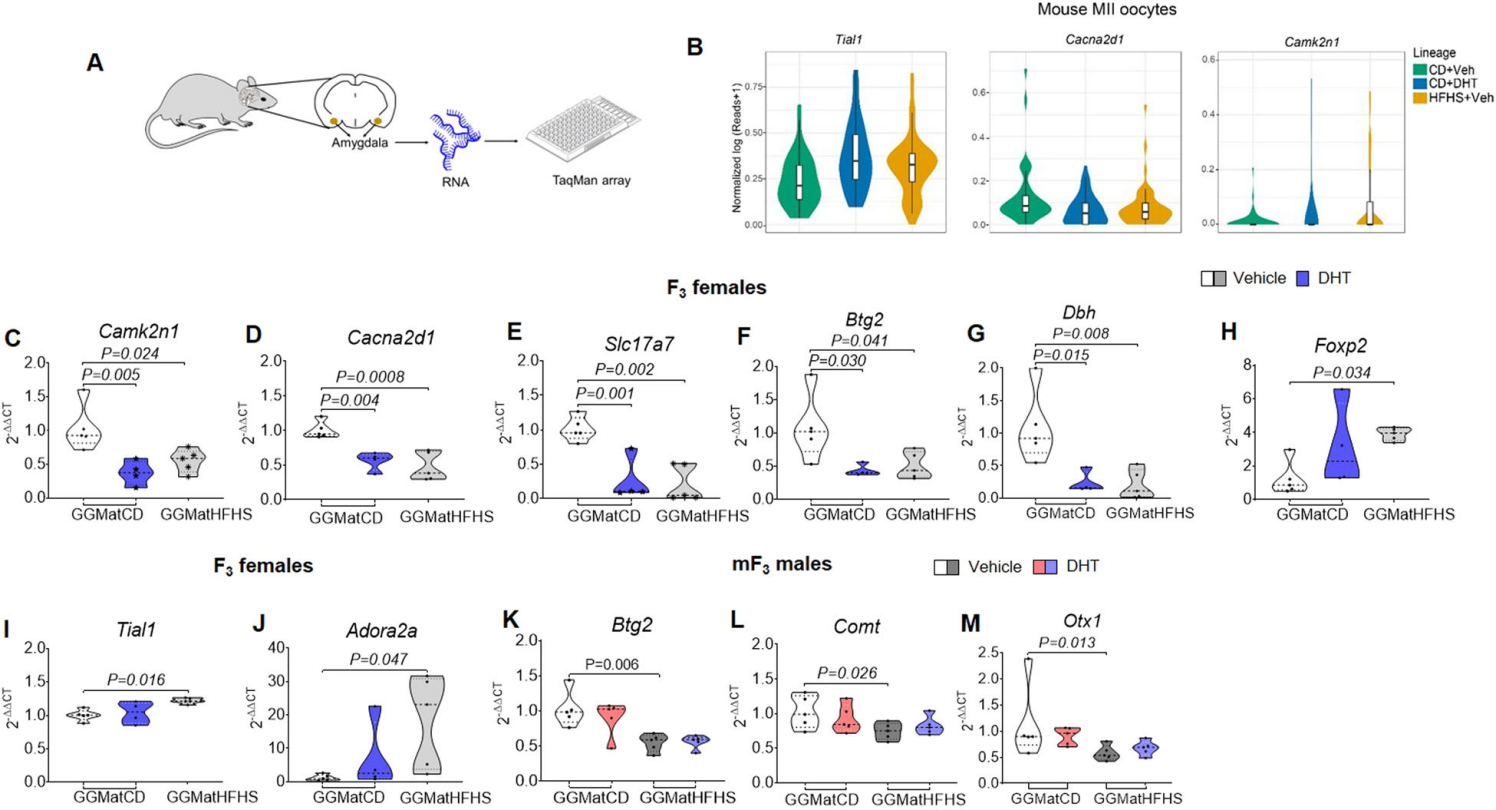
Prenatal androgen exposure with and without maternal obesity causes anxiety-related gene expression changes in the amygdala of F_3_ female and mF_3_ male offspring. **A** Schematic illustration showing low-density TaqMan array of the amygdala. **B** Violin plots showing the expression level of selected DEGs in MII oocytes from each lineage. Violin plots showing the oocyte-specific log-normalized gene expression in the *y*-axis of MII oocytes from each lineage (four animals per lineage; *n* = 42 oocytes in control diet + vehicle, *n* = 48 oocytes in control diet + DHT and *n* = 47 oocytes in HFHS + vehicle). **C**–**M** Violin plots showing on the *y*-axis gene expression by 2^−ΔΔCT^ of **C** *Camk2n1*, **D** *Cacna2d1*, **E** *Slc17a7*, **F** *Btg2*, **G** *Dbh*, **H** *Foxp2*, **I** *Tial1*, **J** *Adora2a*, **K** *Btg2*, **L** *Comt*, and **M** *Otx1* in the amygdala of **C**–**J** F_3_ female offspring (control lineage *n* = 5, androgenized lineage *n* = 4, and obese lineage *n* = 5) and **K**–**M** mF_3_ male offspring (control lineage *n* = 5, androgenized lineage *n* = 5, obese lineage n = 5 and obese and androgenized lineage *n* = 5). The data are present in violin plot (**C**–**J**) showing the frequency distribution curves. The median and quartiles values are shown in dotted and dashed lines, respectively. Two-way ANOVA was followed by Bonferroni post hoc analysis and one-way ANOVA by Bonferroni post hoc analysis. All data are presented as mean ± s.e.m. DHT dihydrotestosterone, CD control diet, HFHS high-fat high-sugar, GGMat great-grand maternal.

As there is a strong indication that prenatal androgen exposure and obesity cause a sexually dimorphic anxiety-like behavior across generations, we performed targeted gene expression analyses within the amygdala of the F_3_ female and the mF_3_ male offspring using a low-density TaqMan array (Fig. 4A and Table S5). Based on our previous single-cell RNA sequencing data of mouse MII oocytes^15^, we selected differentially expressed genes (DEGs) across generations related to anxiety in the androgenized and in the obese lineages (Fig. 4B). In addition, known genes involved in anxiety were selected for analyses^14,16–18^ (Table S5). Several genes related to anxiety showed differential expression in the amygdala in the obese and androgenized lineages of F_3_ female and mF_3_ male offspring. Specifically, in the androgenized and the obese lineages of F_3_ female offspring, we found downregulated expression of the calcium/calmodulin-dependent protein kinase II inhibitor 1 (*Camk2n1*), and the calcium voltage-gated channel auxiliary subunit alpha 2 delta 1 (*Cacna2d1*)^19^ involved in the regulation of Ca^2+^ influx through voltage-gated calcium channels; the solute carrier family 17 member 7 (*Slc17a7*)^20^ involved in glutamate uptake into synaptic vesicles; the dopamine beta-hydroxylase (*Dbh*)^21^ catalyzing the conversion of dopamine to norepinephrine; and the BTG anti-proliferation factor 2 (*Btg2*)^22,23^ involved in neurite outgrowth (Fig. 4C–G). In addition, only in the obese lineage of F_3_ female offspring we found an upregulation of the Forkhead box P2 (*Foxp2*), a transcription factor involved in brain development^24^; the TIA1 cytotoxic granule associated RNA binding protein-like 1 (*Tial1*), involved in synaptic plasticity and the processing of fear memory^25^; and adenosine A2a receptor (*Adora2a*), which influences the fine-tuning of neurotransmitters such as dopamine^26^ (Fig. 4H–J). In mF_3_ male offspring, only the obese lineage showed downregulated expression of the *Btg2*, the Catechol-O-Methyltransferase (*Comt*)^27^ involved in catecholamine metabolism, and the Orthodenticle homeobox 1 (*Otx1*)^28^ involved in brain development (Fig. 4K–M).

### Germ cells versus in utero environment in the transmission of anxiety-like phenotype in offspring

**Fig. 5:**
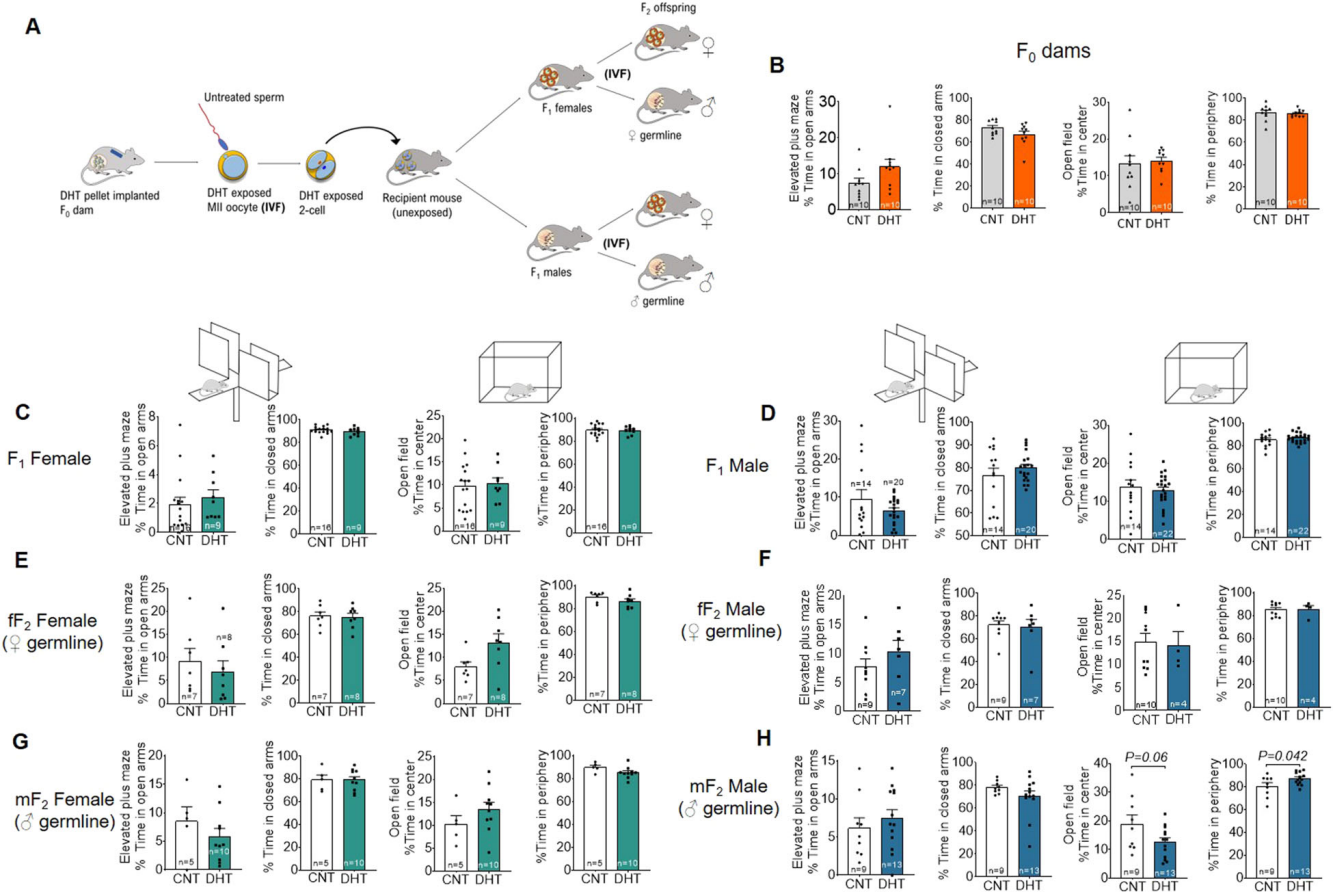
Germ cells versus in utero environment in the transmission of anxiety like phenotype in offspring. **A** Schematic illustration showing prepubertal mouse model and generation of F_1_ and F_2_ offspring by using IVF. **B**–**D** Time spent in the open arms in the elevated plus maze (EPM); time spent in the closed arms of the EPM; time spent in the center of the open filed (OF); time spent in the periphery of the OF. Data were analyzed with Student’s *t*-test. All data are presented as mean ± s.e.m. CNT control, DHT dihydrotestosterone.

Finally, to delineate germ cell factors from the in utero environment in the transmission of anxiety-like phenotype in offspring in PCOS, we used the prepubertal mouse model of PCOS^29^ combined with in vitro fertilization (IVF) and embryo transfer into surrogate mothers (Fig. 5A). Neither F_0_ donors exposed continuously to DHT from puberty until superovulation for oocyte donation (Fig. 5B), nor did the F_1_ female and male offspring display an anxiety-like behavior in the elevated-plus maze and open field tests (Fig. 5C, D). Further, F_2_ female and male offspring in the female germline did not display signs of anxiety-like behavior (Fig. 5E, F). However, following the male germline, the F_2_ male but not the F_2_ female offspring in the DHT lineage displayed an anxiety-like behavior with more time spent in the periphery of the open field (Fig. 5G, H).

Thus, these two sets of experiments in the PNA and prepubertal mouse models of PCOS suggest that the transgenerational transmission of anxiety-like behavior in females following the female germline is likely due to an aberrant health of somatic cells, whereas the transgenerational transmission of anxiety-like behavior in the F_2_ male offspring following the male germline is likely mediated via modifications in the germ cells. The observed male germline effects on anxiety-like behavior is in line with previous studies demonstrating transmission of the epigenetic mark through the male gametes to their offspring^30,31^.

## Discussion

Here, we studied how anxiety disorders are propagated across generations due to maternal PCOS conditions in both human and mouse studies. Our findings showed that daughters, but not sons of women with PCOS are at a 78% higher risk of being diagnosed with anxiety disorders compared with children born to women without PCOS. In a PCOS-like mouse model, prenatal exposure to androgens caused an anxiety-like behavior only in the F_1_ female offspring, which was transgenerationally inherited to the F_3_ female offspring, but not to the male sibling offspring. Maternal obesity led to an increased anxiety-like behavior in F_1_ female offspring, but such effects were not observed in F_3_ offspring via the female germline. In the male germline, mF_3_ males developed anxiety-like behavior in the androgenized and in the obese lineages. Within the amygdala of F_3_ female offspring, five genes (*Camk2n1, Cacna2d1, Slc17a7, Btg2,* and *Dbh*) were downregulated in the androgenized and obese lineages and three genes (*Foxp2, Tial1,* and *Adora2a*) were upregulated only in the obese lineage. Lastly, *Btg2*, *Comt*, and *Otx1* were downregulated within the amygdala of the mF_3_ male offspring in the obese lineage.

Prenatal androgen exposure may cause permanent reprogramming of fetal neural systems thereby increasing the risk of neuropsychiatric disorders^8,32^. In our human and mouse studies, maternal PCOS and in utero androgen exposure, respectively, led to increased anxiety behaviors in F_1_ female offspring, but not in F_1_ male offspring. The mechanism leading to this sexual dimorphic response in behavior is not fully understood, but different hypotheses have been speculated. During the embryonic development, female fetuses are exposed to lower levels of androgens, whereas male fetuses encounter higher levels of androgens from 8th to 24th weeks of gestation as their developing testes also produce androgens^33^. Pregnant women with PCOS have higher levels of androgens throughout pregnancy compared to pregnant women without PCOS^34–40^. Therefore, while both male and female fetuses of women with PCOS encounter higher prenatal androgen levels, it can be hypothesized that the effects on brain development and behavior later in life are stronger in females. Similarly to these observations and our findings, previous studies in animal models of PCOS have shown a sexually dimorphic anxiety-like behavior, with increased anxiety-like behavior in female offspring, which was further supported by sex-specific gene expression changes in the amygdala^13,14,41^. Intrinsic epigenetic differences between the daughters and sons of women with PCOS leading to differential trajectories of DNA methylation in the brain development could also be involved in the observed behavioral sex differences^42,43^. However, studies that investigate epigenetics in the neurodevelopment in PCOS are lacking.

There is accumulating evidence that an adverse in utero environment can influence not only the first generation of offspring but also the subsequent generations^15^. Data from a few epidemiological studies along with studies in animal models have increased our understanding of the effects, and the mechanisms by which changes in the maternal environment during pregnancy can affect the health of children and grandchildren^44,45^. In the current study, we investigated how a maternal environment with androgen excess and obesity affects the behavior of three subsequent generations. Interestingly, we demonstrated that maternal androgen excess had a stronger impact on the transgenerational transmission of anxiety-like behavior in the offspring, rather than maternal obesity. The existing data provide support for transmission of behavioral changes, including anxiety disorders and autism spectrum disorders, in the first generation^9,46–48^, but the evidence is limited for subsequent generations. Other adverse maternal stimuli, such as maternal exposure to glucocorticoids, are shown to increase stress responses and anxiety disorders in the 1st and 2nd generation of offspring, with female offspring being more affected^49,50^.

The transgenerational effects of an adverse maternal environment have been studied more thoroughly than the effects of an adverse paternal environment in the offspring. However, an increasing number of animal studies assess the paternal transmission of effects, providing more accurate information about the involved mechanisms, as many confounding factors are eliminated^45,51^. Paternal transmission of phenotypes is inherited through the genetic and epigenetic material in the sperm, while the maternal transmission may be mediated by germ cells and/or the maternal environment during gestation and lactation. Here, we studied both maternal and paternal transmission of anxiety-like behavior. With regard to the paternal transmission, we showed that the F_1_ male offspring exposed to maternal androgens and/or obesity in utero did not develop behavioral changes, but they did transmit an anxiety-like behavior in the third generation of male offspring (mF_3_) in the androgenized and the obese lineages, respectively. These findings suggest that the observed changes in the mF_3_ offspring may be transmitted through germline inheritance. In the IVF experiment, we showed that mF_2_ male offspring developed an anxiety-like behavior, likely supporting the transmission of anxiety-like behavior through the germ cells. Future experiments investigating epigenetic changes in the sperm are warranted.

The amygdala consists of multiple interconnected nuclei that are involved in emotional processing and play a crucial role in the development of anxiety^13,14,52^. To define the molecular mechanisms underlying the observed transgenerational transmission of anxiety-like behavior in F_3_ female offspring and in mF_3_ male offspring, we analyzed target genes within their amygdala. We found a downregulated expression of genes involved in calcium homeostasis (*Camk2n1*, *Cacna2d1)*, glutamate transport (*Slc17a7*), neurite growth regulator (*Btg2*), and dopamine metabolism (*Dbh*) in both the androgenized and obese lineage of F_3_ female offspring. It has been reported that CAMK2N1 expression is downregulated after androgen receptor (AR) activation in human prostate cancer cell lines following an auto-regulatory negative feedback loop^53,54^. We have also shown a dysregulated expression of *Camk2n1* in the oocytes of the androgenized females. Thus, it can be speculated that *Camk2n1* expression is dysregulated in multiple organs in our prenatally androgenized model due to changes in AR signaling. Calcium voltage-gated channels are important for neurotransmission, and genomic aberrations of the *CACNA2D1* gene have been linked to autism spectrum disorder, epilepsy, and neuropsychiatric disorders^19,55^. Therefore, it can be surmised that prenatal androgenization of F_1_ females resulted in reprogramming of the amygdala and led to dysregulation of genes involved in calcium signaling, among others, causing an anxiety-like behavior in the F_3_ female offspring.

In the male germline, mF_3_ male offspring in the androgenized and obese lineages displayed an anxiety-like behavior. Among the tested genes, none was found dysregulated in the androgenized lineage, but *Comt*, *Btg2*, and *Otx1* were downregulated in the obese lineage. COMT is an enzyme involved in the inactivation of catecholamines, and it has been associated with a broad range of psychiatric phenotypes in human and animal studies^56^. Interestingly, COMT val18met polymorphism has been associated with both anxiety disorders^57^ and abdominal obesity in men, findings that are in line with our data^58^. BTG2 has been shown to interact with AR, by repressing AR transcriptional activity^57^. Other mouse studies have shown downregulation of *Btg2* in the medial prefrontal cortex of male mice with social behavior deficits^59^, and upregulation in the neocortex of mice in response to acute immobilization stress^60^. Finally, *Otx1* is a transcription factor that is evidenced to play an important role in brain development^61^. Mice lacking *Otx1* develop epilepsy and abnormalities in many brain regions^62^.

Our recent publication shows that androgen is the main driver and likely reprograms germ cells and/or adversely affects the in utero environment, propagating a PCOS-like phenotype up to three generations. Here, we extend these findings and confirm that an anxiety-like phenotype is transgenerationally transmitted only in the androgenized lineage in females, whereas it is transmitted to the third generation in both the obese and androgenized lineages in mF_3_ male offspring. To dissect germ cell factors from the in utero environment in the transmission of anxiety-like phenotype in PCOS female and male offspring, we used the prepubertal mouse model of PCOS and combined it with IVF and embryo transfer to a healthy surrogate mother to generate F_1_ and F_2_ offspring. Intriguingly, the female offspring did not display anxiety-like behavior. On the other hand, mF_2_ male offspring in the androgenized lineage displayed anxiety-like behavior demonstrating that androgen exposed oocytes carry reprogrammed information and transmit it transgenerationally to the mF_2_ male offspring.

Although differences do exist, the circuitry and function of the amygdala are well-conserved across species^63,64^. Therefore, our rodent behavioral experiments and subsequent molecular analyses provide some clues to the potential pathways behind the increased risk for anxiety diagnoses found in the daughters of women with PCOS.

Two other studies have investigated anxiety in the children born to women with PCOS. Robinson et al.^11^ reported a 62% increased risk of a childhood anxiety diagnosis when compared to children born to women without PCOS. This study was conducted in a US population-based birth cohort, where both the PCOS status of the mother and any childhood anxiety diagnosis was reported by the mother when the children 7 or 8 years old. Chen et al.^12^ used a Finnish population-based cohort study and reported a 33% increased risk (HR 1.33; 95% CI 1.26–1.41) for an anxiety diagnosis in children born to women with PCOS, however they did not find a difference for male and female children. Comparatively, we report a slightly higher risk for anxiety overall, and specifically an increased risk for daughters born to women with PCOS.

The strength in our study lies in the large study size due to the use of health register data covering the entire population in Sweden. Additionally, data on diagnoses of both PCOS and anxiety were reported from hospital-based specialist care to the registries, and we had a long follow-up time for the children. Nonetheless, there are some inherent limitations to observational register-based data. The prevalence of anxiety amongst the children in the study population was lower than previously reported^65^, likely because we do not include diagnoses made in primary care, and possibly because the study population is younger than the estimated mean age at onset of anxiety (21.3 years)^66^. Further, it is well established that women with PCOS have increased rates of anxiety^7^. In our study, while we accounted for parental history of psychiatric disorders in the analysis, we were unable to investigate separately the role of genetic (i.e., related to anxiety and behavior) and environmental factors (e.g., prenatal androgen exposure, maternal stress) which could influence the risk of anxiety disorders in the children. The mothers with PCOS in the study had elevated BMI compared to mothers without PCOS. However, due to differential missingness of the BMI information, we avoided biasing our results by not including BMI in the main analysis models. Instead we conducted a complete case analysis, and despite the slight difference between the subsample and the full cohort, the estimates followed a similar pattern as in the main analysis albeit with attenuated HRs and wider confidence intervals due to the lower numbers. However, this strengthens the findings in the main analysis, as the association is found is not completely attributable to maternal BMI. Similarly, Chen et al.^12^ found that their results suggested a synergistic effect of PCOS and obesity on offspring anxiety. This agrees with the mouse model experiments where the influence of maternal obesity was explored. Further, due to the young age of the cohort, we were unable to assess any transgenerational effects of maternal PCOS on subsequent generations. The strength of our study is that we translate our preclinical mice data to real-life clinical data.

In summary, we here show that daughters of women with PCOS are more likely to develop anxiety disorders, but not sons. In a mouse model of PCOS, we revealed that maternal androgen excess causes transgenerational transmission of anxiety-like behavior in female offspring (F_1_ and F_3_), but not in F_1_, F_2_, and F_3_ male siblings. We further demonstrated that the first generation of unaffected male offspring exposed to maternal androgens and maternal obesity did also transmit an anxiety-like behavior in subsequent male generations (mF_3_). These behavioral changes in the F_3_ female offspring and mF_3_ male offspring were accompanied by gene expression changes in the amygdala. Our findings provide evidence that elevated maternal androgens in PCOS accompanied by maternal obesity may underlie the risk of transgenerational transmission of anxiety disorders in daughters and sons of women with PCOS, an effect that can be mediated by epigenetic reprogramming.

## Materials and methods

### Ethical approvals

The authors assert that all procedures contributing to this work comply with the ethical standards of the relevant national and institutional committees on human experimentation and with the Helsinki Declaration of 1975, as revised in 2008. The cohort study was approved by the regional ethical review board in Stockholm, Sweden (diary number 2013/862-31/5; 2016/1214-32). The requirement for informed consent was waived because the study was register-based, and the included individuals were not identifiable at any time.

Animal experiments were done in accordance with the legal requirements of the European Community (SJVFS 2017:40) and the directive 2010/63/EU of the European Parliament on the protection of animals used for scientific purposes and approved by the Stockholm Ethical Committee for Animal Research (Dnr. No. 10798-2017). Animal care and procedures were controlled by Comparative Medicine Biomedicum (KM-B), Karolinska Institutet, Stockholm, Sweden.

### Register-based cohort study population

Using the unique personal identification number assigned to each individual in Sweden at birth or at immigration, several nationwide longitudinal registers containing health and sociodemographic data until December 31, 2013, were linked, including the Swedish Medical Birth Register (MBR), National Patient Register (NPR), Prescribed Drug Register (PDR), Total Population Register (TPR), Migration Register, and the Cause of Death Register (CDR).

Women who had delivered at least one child were identified through the MBR. The NIH diagnostic criteria for PCOS were established in 1990 followed by the Rotterdam criteria 2003^67^. Women with PCOS were identified by having at least one PCOS International Classification of Diseases (ICD) code (ICD-9: 256E; ICD-10: E28.2) recorded in the MBR or in the NPR after the age of 13 and between 1990 and 2013. Women with a concurrent diagnosed condition that could cause symptoms similar to PCOS were excluded to ensure specificity^4,8^. This yielded a total of 12,955 mothers with PCOS. Time of PCOS diagnosis (before or after pregnancy) was not taken into account since elevated testosterone levels have been reported to be present throughout life in women with PCOS^5^. Each mother with PCOS was then matched to upto ten comparison mothers without a PCOS diagnosis randomly selected from the general population on her birth year and county of residence within the year of diagnosis.

Children born from 1995 to 2007 to mothers with PCOS and matched unaffected mothers were identified. All children from each mother were included. Children were excluded if they were born outside of Sweden, adopted, stillborn or died on the day of birth, or had congenital malformations. This yielded a total of 8864 children born to women with PCOS and 89,431 children born to women without PCOS from the general population. The birth years of 1995 to 2007 were chosen so that the children had complete coverage in the outpatient register (which began in 2001) from the age-eligible for anxiety diagnoses (age 6). Children were followed until December 31, 2013, yielding an age range of 6–18.9 years at the end of follow-up.

#### Outcome classification

Diagnoses of anxiety recorded after the age of 6 were identified by ICD-10 codes F40.0, F40.1, F41.0, F41.1, and F43.

#### Covariates

Highest attained maternal education level (primary and secondary education, upper secondary education, post-secondary/post-graduate education) was used as a proxy for the child’s socioeconomic status. Maternal and paternal lifetime history of psychiatric disorders was determined by any recorded psychiatric diagnosis in the NPR. Region of birth (Nordic/non-Nordic) was extracted from the TPR. The child’s sex and year of birth were extracted from the MBR. Data on maternal body mass index (BMI) was available in the MBR, however, due to a high proportion of missing values (13%) combined with data not missing at random (missingness was associated with the outcome) it was not included in the main analysis so as not to introduce bias.

### Animals

The number of mice used for behavioral testing and for gene expression analyses is given in the figure legend and/or text. Details of breeding of females and male sibling have previously been described^15^ and is detailed in Table S4. In brief, 21-days-old C57Bl/6J mice were obtained from Janvier Labs (Le Genest-Saint-Isle, France). All mice were maintained under a 12-h light/dark cycle and in a temperature-controlled room with ad libitum access to water and a diet. After one week of acclimatization, female mice were randomly divided into two groups and fed either 1) a CD (Research Diets, D12328) comprising 11% fat, 73% carbohydrates [0% sucrose], and 16% proteins or 2) HFHS diet (Research Diets, D12331) comprising 58% fat, 26% carbohydrates [17% sucrose], and 16% proteins) for six weeks. A female in the proestrus or estrus phase, determined by vaginal cytology, was mated overnight with a male. Females were checked daily for post-copulatory plugs, and a plug on the morning after mating was considered embryonic day (E) 0.5. Details of the number of animals used for 1) phenotypic testing and 2) for breeding to generate F_1_, F_2_, and F_3_ in each group have previously been described^15^.

### Prenatal androgen exposure

The CD and HFHS groups were in random order subdivided and subjected to daily injections subcutaneously (s.c.) in the interscapular area from E16.5 to E18.5 with 50 µl of a solution containing: 1) a mixture of 5 µl benzyl benzoate (B6630; Sigma-Aldrich) and 45 µl sesame oil (S3547; Sigma-Aldrich, St. Louis, Missouri, USA) i.e., vehicle, or 2) 250 µg DHT (5α androstane-17β-ol-3-one, A8380; Sigma-Aldrich, St. Louis, Missouri, USA) dissolved in a mixture of 5 µl benzyl benzoate and 45 µl sesame oil i.e., prenatal androgen exposure by DHT. In brief, prior to mating with male mice, F_0_ mothers were fed CD or HFHS-diet for 6 weeks^15^. During the embryonic day (E)16.5–E18.5 pregnant mice were injected subcutaneously with dihydrotestosterone (DHT), a non-aromatizable androgen, or sesame oil alone (vehicle) resulting in four experimental groups; MatCD+Vehicle (control), MatCD+DHT (androgenized), MatHFHS+Vehicle (obese), and MatHFHS+DHT (obese and androgenized). These are well-established models of prenatal androgen exposure and diet-induced obesity^14,15^. F_1_ female offspring were mated with unrelated healthy males fed CD to generate F_2_ and thereafter to generate F_3_ female and male (siblings) offspring (female germline) (Fig. 2A). Furthermore, F_1_ males were mated with unrelated health females fed CD to generate mF_2_ and mF_3_ males (male germline) (Fig. 2A and Table S4). Anxiety-like behavior was assessed in the elevated plus maze and in the open field at adult age in F_1_, F_2_ and F_3_ female offspring and in their F_2_ and F_3_ male siblings (fF_2_ and fF_3_), as well as in F_1_, mF_2_ and mF_3_ male offspring. Compromised embryonic development of second-generation (F_2_) offspring in the obese and androgenized female lineage^15^ prevented us from investigating the transgenerational transmission of anxiety-like behavior in F_3_ offspring in the combined lineage.

### Mouse-breeding scheme and feeding paradigm to generate F_1_ to F_3_ generations

All offspring, F_1_, F_2,_ and F_3_ were weaned at postnatal day 21 onto control diet: CD+Veh, CD+DHT, HFHS+Veh, and HFHS+DHT. To generate F_2_, a subset of F_1_ female and male offspring were mated with unrelated males and females fed the control diet, respectively, and a subset of F_2_ female and male offspring were mated with unrelated males and females, respectively, to generate F_3_. Remaining F_1_, F_2_, and F_3_ female and male offspring were subjected to phenotypic behavioral testing as described below.

### Prepubertal androgen exposure, in vitro fertilization, and offspring generation

To generate the prepubertal PCOS-like mouse model, four week-old female mice were implanted subcutaneously with a 10-mm length pellet of crystalline 5α-DHT (A8380-1G; Sigma-Aldrich, St. Louis, MO) and as control, a non-DHT containing pellet was inserted^29^. After 6 weeks of pellet implantation, F_0_ donors were subjected to phenotypic behavioral testing. Then these female mice (10-week of age) were superovulated and oocytes were collected and added to capacitated spermatozoa to perform in vitro fertilization (IVF) in the Karolinska Center for Transgene Technologies (KCTT), Karolinska Institutet. Two-cell embryos were transferred into healthy surrogate mothers to generate F_1_ female and male offspring. A subset of F_1_ female and male offspring, respectively, underwent IVF to generate F_2_ female and male offspring thus following both female and male germline. F_0_ donors and remaining F_1_ and F_2_ female and male offspring were subjected to phenotypic behavioral testing as described below.

### Assessment of behavior

All behavior experiments were performed at age of 16–17 weeks and during the light phase of the light-dark cycle, and mice were acclimatized in the testing room for 20-min before the test. In the EPM and OF tests, the mice were tracked automatically by an infrared digital camera using the EthoVision XT software (Noldus, Wageningen, the Netherlands). Innate fear/anxiety was assessed by using elevated plus maze as previously described^14^. Briefly, mice were placed into the middle of a four-armed maze facing an open arm and allowed to explore for 10-min. The number of arm entries and distance covered was tracked and analyzed by EthoVisionXT Software. We assessed anxiety-like behavior by calculating the percentage of time spent in the open and closed arms. For open field, the mouse was placed at the center of the arena and its movements were recorded for 10 min. We evaluated anxiety-like behavior and general locomotor activity by calculating the percentage of time spent in the center and the total distance traveled, respectively. The OF test was performed 4 days after the EPM.

### Statistical analysis

In the register-based cohort, children were followed from age 6, when they were eligible to receive a diagnosis of anxiety, to the date of diagnosis, death, emigration, age 18, or end of follow-up (December 31, 2013), whichever came first. The mean age and standard deviation at the end of follow-up were 11.2 ± 3.6 years. Associations between maternal PCOS and offspring anxiety were estimated as hazard ratios (HR) with 95% confidence intervals (CI) using stratified Cox regression models with attained age as the underlying time scale. In addition to the maternal matching criteria (maternal birth year and county of residence within the year of PCOS diagnosis), potential confounding variables were adjusted for including offspring sex and year of birth, maternal age at child’s birth, maternal education, maternal region of birth, and maternal and paternal lifetime history of psychiatric disorders (adjusted Model). Robust standard errors were used to account for dependence between observations since several children from the same family were included in the study population. The analysis was conducted first for all offspring combined, then stratified by offspring sex. A sensitivity analysis including only the subset of the population with BMI data was conducted.

In the mice experiments, data were assessed for normality and variance (Kolmogorov Smirnov). Group allocation during experiments was not blinded to the investigators. However, data analyses were repeated by two or more investigators. The sample size in the mice experiments was based on differences in AGD between the control and the androgenized lineage in our previous studies^14,68^. Nine animals per group were required to detect a mean difference in AGD of 40.6% with a standard deviation (SD) of 0.1, a significance level of 0.05, and a power of 0.8. All data are presented as mean ± s.e.m, SD or as median and range. Differences between groups were determined by two-way ANOVA followed by Tukey’s post hoc test or Bonferroni post hoc test, and by one-way ANOVA followed by Tukey’s post hoc test or Bonferroni post hoc test. For the IVF experiment, we performed Student’s *t*-test in normalized data. Differences were considered statistically significant at *P* < 0.05. Statistical analyses for the register-based cohort study were performed using Stata statistical software version 15.1 (Stata Corps, Texas, USA), and in the animal studies by GraphPad Prism 8 (GraphPad Software Inc., CA, USA) and SPSS software v.26.0 (IBM, Armonk, NY, USA).

## Supplementary information

Fig. S1

Table S1

Table S2

Table S3

Table S4

Table S5

AJ Checklist

## Data Availability

All data associated with this study are presented in the paper or supplementary materials.
