# Supplementary figures and images for: Prenatal androgen exposure causes a sexually dimorphic transgenerational increase in offspring susceptibility to anxiety disorders
Source: Transl Psychiatry. 2021 Jan 13;11:45. doi: 10.1038/s41398-020-01183-9 (PMC7806675; doi:10.1038/s41398-020-01183-9)

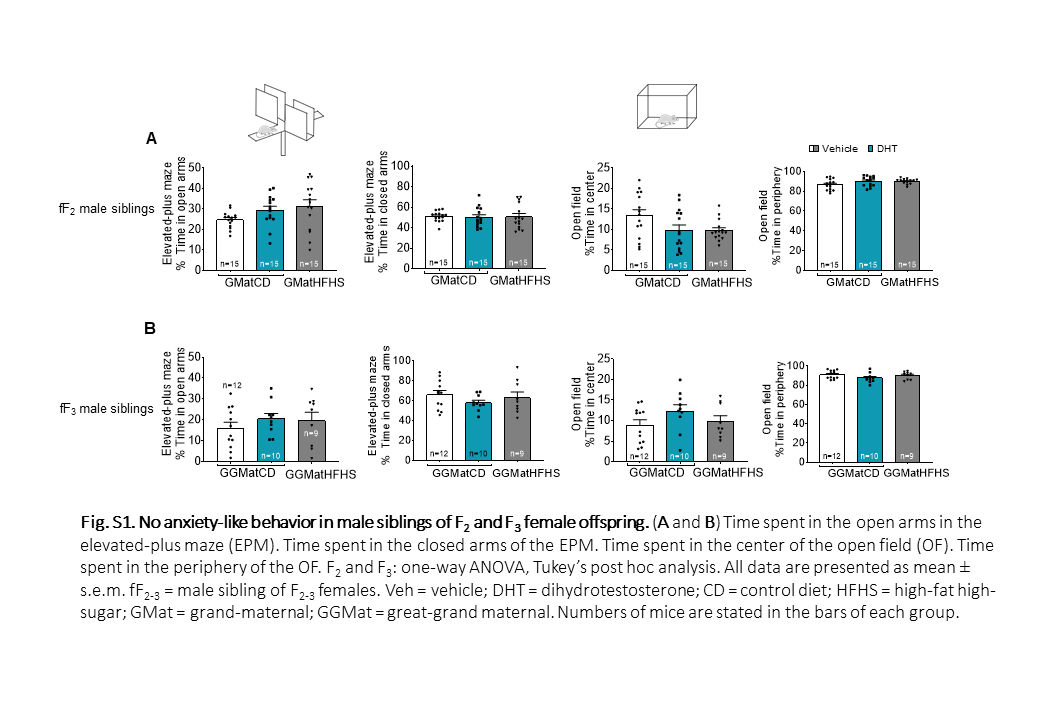

Supplement: Supplementary file 1 — Fig. S1 [file 41398_2020_1183_MOESM1_ESM.tif]
