# Supplementary material for: Prenatal androgen exposure causes a sexually dimorphic transgenerational increase in offspring susceptibility to anxiety disorders
Source: Transl Psychiatry. 2021 Jan 13;11:45. doi: 10.1038/s41398-020-01183-9 (PMC7806675; doi:10.1038/s41398-020-01183-9)
Supplement: Supplementary file 2 — Table S1 [file 41398_2020_1183_MOESM2_ESM.docx]

**Table S1:** Characteristics of study population: children born 1995 to 2007.

|  | **Children born to women without PCOS**  **n=93602** | | **Children born to women with PCOS**  **n=8864** | | **All Children**  **n=102466** | |
| --- | --- | --- | --- | --- | --- | --- |
|  | n | (%) | n | (%) | n | (%) |
| **Child's sex** | | | | | | |
| Boys | 47806 | 51.07 | 4574 | 51.6 | 52380 | 51.12 |
| Girls | 45796 | 48.93 | 4290 | 48.4 | 50086 | 48.88 |
| **Maternal age at child’s birth (years)** | | | | | | |
| <20 | 2943 | 3.14 | 250 | 2.82 | 3193 | 3.12 |
| 20-24 | 20950 | 22.38 | 1789 | 20.18 | 22739 | 22.19 |
| 25-29 | 37625 | 40.2 | 3206 | 36.17 | 40831 | 39.85 |
| 30-34 | 25783 | 27.55 | 2689 | 30.34 | 28472 | 27.79 |
| 35+ | 6301 | 6.73 | 930 | 10.49 | 7231 | 7.06 |
| **Maternal region of birth** | | | | | | |
| The Nordic countries | 77887 | 83.21 | 7041 | 79.43 | 84928 | 82.88 |
| Outside the Nordic countries | 15715 | 16.79 | 1823 | 20.57 | 17538 | 17.12 |
| **Maternal highest achieved education** | | | | | | |
| Primary and secondary education | 9067 | 9.69 | 961 | 10.84 | 10028 | 9.79 |
| Upper secondary education | 43021 | 45.96 | 4163 | 46.97 | 47184 | 46.05 |
| Post-secondary/post-graduate education | 41168 | 43.98 | 3725 | 42.02 | 44893 | 43.81 |
| missing | 346 | 0.37 | 15 | 0.17 | 361 | 0.35 |
| **Maternal pre-pregnancy BMI (**kg/m^2^**)** | | | | | | |
| Below 25 | 52883 | 56.50 | 3033 | 34.22 | 55916 | 54.57 |
| 25 and above | 28281 | 30.21 | 4734 | 53.41 | 33015 | 32.22 |
| missing | 12438 | 13.29 | 1097 | 12.38 | 13535 | 13.21 |
| **Maternal lifetime history of psychiatric diagnosis** | | | | | | |
| None | 78785 | 84.17 | 6829 | 77.04 | 85614 | 83.55 |
| Any | 14817 | 15.83 | 2035 | 22.96 | 16852 | 16.45 |
| **Paternal lifetime history of psychiatric diagnosis** | | | | | | |
| None | 83291 | 88.98 | 7776 | 87.73 | 91067 | 88.88 |
| Any | 10311 | 11.02 | 1088 | 12.27 | 11399 | 11.12 |
| **Child’s Anxiety diagnosis** | | | | | | |
| No | 92690 | 99.03 | 8747 | 98.68 | 101437 | 99.0 |
| Yes | 912 | 0.97 | 117 | 1.32 | 1029 | 1.0 |

Abbreviations: BMI: body mass index, PCOS: polycystic ovary syndrome.
