# Supplementary material for: Prenatal androgen exposure causes a sexually dimorphic transgenerational increase in offspring susceptibility to anxiety disorders
Source: Transl Psychiatry. 2021 Jan 13;11:45. doi: 10.1038/s41398-020-01183-9 (PMC7806675; doi:10.1038/s41398-020-01183-9)
Supplement: Supplementary file 3 — Table S2 [file 41398_2020_1183_MOESM3_ESM.docx]

**Table S2:** Crude and adjusted hazard ratios and 95% confidence intervals for the risk of anxiety diagnoses in children born to women with and without PCOS.

|  | **All Children**  **HR (95% CI)** | **Boys**  **HR (95% CI)** | **Girls**  **HR (95% CI)** |
| --- | --- | --- | --- |
| Number of exposed children diagnosed with anxiety | 117 (1.32%) | 38 (0.83%) | 79 (1.84%) |
| Crude model | 1.74 (1.35-2.25) | 1.42 (0.89-2.25) | 2.14 (1.47-3.13) |
| Adjusted Model 1 | 1.49 (1.16-1.92) | 1.15 (0.71-1.86) | 1.78 (1.19-2.67) |

Abbreviations: CI = confidence interval; HR = hazard ratio; PCOS = polycystic ovary syndrome.

Adjusted Model 1: sex of child*, maternal age, maternal country of birth, maternal education, maternal and paternal psychiatric history, and year of birth of child.

*sex was excluded from the sex stratified models
