# Supplementary material for: Prenatal androgen exposure causes a sexually dimorphic transgenerational increase in offspring susceptibility to anxiety disorders
Source: Transl Psychiatry. 2021 Jan 13;11:45. doi: 10.1038/s41398-020-01183-9 (PMC7806675; doi:10.1038/s41398-020-01183-9)
Supplement: Supplementary file 4 — Table S3 [file 41398_2020_1183_MOESM4_ESM.docx]

**Table S3**: BMI Complete Case Analysis: Crude and adjusted hazard ratios and 95% confidence intervals for the risk of anxiety diagnoses in children born to women with and without PCOS.

|  | All Children  HR (95% CI) | Boys  HR (95% CI) | Girls  HR (95% CI) |
| --- | --- | --- | --- |
| Crude model | 1.56 (1.18-2.07) | 1.41 (0.85-2.34) | 1.89 (1.24-2.89) |
| Adjusted Model 1 | 1.34 (1.00-1.78) | 1.18 (0.70-1.98) | 1.53 (0.96-2.42) |

Adjusted Model 1: sex of child*, maternal age, maternal country of birth, maternal education, maternal and paternal psychiatric history, year of birth of child and BMI.

*sex was excluded from the sex stratified models
