# Supplementary material for: Prenatal androgen exposure causes a sexually dimorphic transgenerational increase in offspring susceptibility to anxiety disorders
Source: Transl Psychiatry. 2021 Jan 13;11:45. doi: 10.1038/s41398-020-01183-9 (PMC7806675; doi:10.1038/s41398-020-01183-9)
Supplement: Supplementary file 5 — Table S4 [file 41398_2020_1183_MOESM5_ESM.docx]

**Table S4.** Details of number of animals used in experiment for a) phenotypic testing and b) for breeding to generate F_1_, F_2_ and F_3_ in each group.

**F_0_ → F_1_ for the offspring study**

| **Group** | Number of F_0_ before mating | Pregnant: F_0_ breeders | Female: F_1_ offspring | Male: F_1_ offspring | F1 female + F_1_ male used for phenotyping (number of F0 breeders) |
| --- | --- | --- | --- | --- | --- |
| CD+Veh | 40 | 27 | 58^#^ | 54 | 15+10 (15) |
| CD+DHT | 40 | 35 | 46 | 41 | 15+10 (11) |
| HFHS+Veh | 40 | 27 | 43 | 42 | 13+10 (10) |
| HFHS+DHT | 40 | 36 | 40* | 50 | 12+10 (8) |
| ^#^ 3 female pups died; * 2 female pups died all due to teeth problems | | | | | |

**F_1_ → F_2_ for the offspring study**

| **Group** | Number  of F_1_ in breeding (number of F_0_ breeders) | Pregnant: F_1_ breeders | Female: F_2_ | Male: F_2_ | F_2_ female+fF_2_ male used for phenotyping  (number of F_1_ breeders) |
| --- | --- | --- | --- | --- | --- |
| CD+Veh | 37 (12) | 11 | 22 | 31 | 13+15 (6) |
| CD+DHT | 31 (24) | 6 | 16 | 15 | 11+15 (3) |
| HFHS+Veh | 29 (17) | 18 | 25 | 28 | 13+15 (7) |
| HFHS+DHT | 24 (28) | 12 | 1 | 0 | 1 (1) |

**F_2_ → F_3_ for the offspring study**

| **Group** | Number  of F_2_ in breeding  (nr of F1 breeders) | Pregnant:  F_2_ breeders | Female: F_3_ | Male: fF_3_ | F_3_ female + fF3 male used for phenotyping (number of F2 breeders) |
| --- | --- | --- | --- | --- | --- |
| CD+Veh | 6 (5) | 6 | 11 | 19 | 11+12 (5) |
| CD+DHT | 4 (3) | 4 | 8 | 10 | 8+10 (4) |
| HFHS+Veh | 9 (11) | 6^¤^ | 9 | 9 | 9+9 (5) |
| HFHS+DHT | 1 (1) | 1 | 0 | 2 (died at postnatal day 18) | 0 |
| ^¤^ 1 female delivered dead pups | | | | | |

**F_1_ → mF_2_ for the offspring study**

| **Group** | Number of F_1_ male in breeding | Male: mF_2_ | mF_2_ male used for phenotyping |
| --- | --- | --- | --- |
| CD+Veh | 4 | 17 | 12 |
| CD+DHT | 4 | 14 | 14 |
| HFHS+Veh | 4 | 18 | 16 |
| HFHS+DHT | 4 | 17 | 16 |

**mF_2_ → mF_3_ for the offspring study**

| **Group** | Number of F_1_ male in breeding | Male: mF_3_ | mF_3_ male used for phenotyping |
| --- | --- | --- | --- |
| CD+Veh | 4 | 16 | 16 |
| CD+DHT | 4 | 23 | 15 |
| HFHS+Veh | 4 | 19 | 13 |
| HFHS+DHT | 4 | 14 | 12 |
