# Supplementary material for: Prenatal androgen exposure causes a sexually dimorphic transgenerational increase in offspring susceptibility to anxiety disorders
Source: Transl Psychiatry. 2021 Jan 13;11:45. doi: 10.1038/s41398-020-01183-9 (PMC7806675; doi:10.1038/s41398-020-01183-9)
Supplement: Supplementary file 6 — Table S5 [file 41398_2020_1183_MOESM6_ESM.pdf]

Table S5: Selection of genes analyzed by the low-density TaqMan array in amygdala of F^1 and mF^3 male offspring.

S.No	Genes	Enterz ID	DOI/References	Functions
				**Calcium Signaling**
1	*Camk2n1*	55450		[illegible] Coding gene.Potent and specific inhibitor of CaM-kinase II (CAMK2).
2	*Cacna2d1*	781	https://doi.org/10.1186/s13229-017-0160-x	CACNA2D1 (Calcium Voltage-Gated Channel Auxiliary Subunit Alpha2delta 1) is a Protein Coding gene. The alpha-2/delta subunit of voltage-dependent calcium channels regulates calcium current density and activation/inactivation kinetics of the calcium channel. Plays an important role in excitation-contraction coupling
3	*Ryr2*	6262	https://doi.org/10.1186/s13229-017-0160-x	RYR2 (Ryanodine Receptor 2) is a Protein Coding gene.Biased expression in heart (RPKM 47.0) and brain (RPKM 4.2). This gene encodes a ryanodine receptor found in cardiac muscle sarcoplasmic reticulum. The encoded protein is one of the components of a calcium channel, composed of a tetramer of the ryanodine receptor proteins and a tetramer of FK506 binding protein 1B proteins, that supplies calcium to cardiac muscle. Mutations in this gene are associated with stress-induced polymorphic ventricular tachycardia and arrhythmogenic right ventricular dysplasia. [provided by RefSeq, Jul 2008]
4	*Disc1*	27185	https://www.ncbi.nlm.nih.gov/pubmed/29212023; https://doi.org/10.1038/mp.2016.231	DISC1 (DISC1 Scaffold Protein) is a Protein Coding gene.Involved in the regulation of multiple aspects of embryonic and adult neurogenesis. Required for neural progenitor proliferation in the ventrical/subventrical zone during embryonic brain development and in the adult dentate gyrus of the hippocampus. Participates in the Wnt-mediated neural progenitor proliferation as a positive regulator by modulating GSK3B activity and CTNNB1 abundance. Plays a role as a modulator of the AKT-mTOR signaling pathway controlling the tempo of the process of newborn neurons integration during adult neurogenesis, including neuron positioning, dendritic development and synapse formation. Inhibits the activation of AKT-mTOR signaling upon interaction with CCDC88A. Regulates the migration of early-born granule cell precursors toward the dentate gyrus during the hippocampal development. Plays a role, together with PCNT, in the microtubule network formation.
				**GABA and Glutamate Metabolism and Receptors**
5	*Gria4*	2893	https://doi.org/10.1038/s41398-018-0258-8.	GRIA4 (Glutamate Ionotropic Receptor AMPA Type Subunit 4) is a Protein Coding gene.AMPA receptors are members of the ionotropic class of glutamate receptors, which
6	*Slc17a6*	57084	https://doi.org/10.1016/j.neuron.2017.09.026	SLC17A6 (Solute Carrier Family 17 Member 6) is a Protein Coding gene. Mediates the uptake of glutamate into synaptic vesicles at presynaptic nerve terminals of excitatory neural cells. May also mediate the transport of inorganic phosphate.
7	*Slc17a7*	57030	https://doi.org/10.1016/j.neuron.2017.09.026	SLC17A7 (Solute Carrier Family 17 Member 7) is a Protein Coding gene. Mediates the uptake of glutamate into synaptic vesicles at presynaptic nerve terminals of excitatory neural cells. May also mediate the transport of inorganic phosphate.
8	*Gad1*	2571	https://doi.org/10.1016/j.neuron.2017.09.026	GAD1 (Glutamate Decarboxylase 1) is a Protein Coding gene. Catalyzes the production of GABA.
9	*Gad2*	2572	https://doi.org/10.1016/j.neuron.2017.09.026	GAD2 (Glutamate Decarboxylase 2) is a Protein Coding gene. Catalyzes the production of GABA

10	*Slc32a1*	140679	https://doi.org/10.1016/j.neuron.2017.09.026	SLC32A1 (Solute Carrier Family 32 Member 1) is a Protein Coding gene. Involved in the uptake of GABA and glycine into the synaptic vesicles.
11	*Gabbr1*	2550	https://doi.org/10.1186/s13148-017-0408-5	GABBR1 (Gamma-Aminobutyric Acid Type B Receptor Subunit 1) is a Protein Coding gene.This gene encodes a receptor for gamma-aminobutyric acid (GABA), which is the main inhibitory neurotransmitter in the mammalian central nervous system. This receptor functions as a heterodimer with GABA(B) receptor 2. Defects in this gene may underlie brain disorders such as schizophrenia and epilepsy. Alternative splicing generates multiple transcript variants, but the full-length nature of some of these variants has not been determined.
	**Glucocorticoid Receptors**			
12	*Crhr2*	1395	https://doi.org/10.1096/fj.201701263RR	CRHR2 (Corticotropin Releasing Hormone Receptor 2) is a Protein Coding gene.G-protein coupled receptor for CRH (corticotropin-releasing factor), UCN (urocortin), UCN2 and UCN3. Has high affinity for UCN. Ligand binding causes a conformation change that triggers signaling via guanine nucleotide-binding proteins (G proteins) and down-stream effectors, such as adenylate cyclase. Promotes the activation of adenylate cyclase, leading to increased intracellular cAMP levels.
	**Androgen-Estrogen signaling**			
13	*Esr1*	2099	https://doi.org/10.1016/j.neuron.2017.09.026	ESR1 (Estrogen Receptor 1) is a Protein Coding gene. Estrogen controls many cellular processes including growth, differentiation and function of the reproductive system. Estrogen is also responsible for the growth and maintenance of the skeleton and the normal function of the cardiovascular and nervous systems.
14	*Esr2*	2100	https://doi.org/10.3389/fpsyt.2019.00081	ESR2 (Estrogen Receptor 2) is a Protein Coding gene. Estrogen controls many cellular processes including growth, differentiation and function of the reproductive system. Estrogen is also responsible for the growth and maintenance of the skeleton and the normal function of the cardiovascular and nervous systems.
15	*Btg2*	7832	https://doi.org/10.1016/j.bbi.2019.03.006.	BTG2 (BTG Anti-Proliferation Factor 2) is a Protein Coding gene. Anti-proliferative protein; the function is mediated by association with deadenylase subunits of the CCR4-NOT complex. Activates mRNA deadenylation in a CNOT6 and CNOT7-dependent manner. In vitro can inhibit deadenylase activity of CNOT7 and CNOT8. Involved in cell cycle regulation. Could be involved in the growth arrest and differentiation of the neuronal precursors (By similarity). Modulates transcription regulation mediated by ESR1. Involved in mitochondrial depolarization and neurite outgrowth.
16	*Ar*	367	https://doi.org/10.1073/pnas.1507514112	AR (Androgen Receptor) is a Protein Coding gene.Steroid hormone receptors are ligand-activated transcription factors that regulate eukaryotic gene expression and affect cellular proliferation and differentiation in target tissues. Transcription factor activity is modulated by bound coactivator and corepressor proteins like ZBTB7A that recruits NCOR1 and NCOR2 to the androgen response elements/ARE on target genes, negatively regulating androgen receptor signaling and androgen-induced cell proliferation (PubMed:20812024). Transcription activation is also down-regulated by NR0B2. Activated, but not phosphorylated, by HIPK3 and ZIPK/DAPK3

17	*Srd5a2*	6716	https://www.ncbi.nlm.nih.gov/pubmed/22776423; https://www.ncbi.nlm.nih.gov/pmc/articles/PMC101357/	SRD5A2 (Steroid 5 Alpha-Reductase 2) is a Protein Coding gene.Converts testosterone (T) into 5-alpha-dihydrotestosterone (DHT) and progesterone or corticosterone into their corresponding 5-alpha-3-oxosteroids. It plays a central role in sexual differentiation and androgen physiology.
18	*Tfap2c*	7022	https://doi.org/10.1095/biolreprod.114.126474; https://www.ncbi.nlm.nih.gov/pmc/articles/PMC5888809/	TFAP2C (Transcription Factor AP-2 Gamma) is a Protein Coding gene.Sequence-specific DNA-binding protein that interacts with inducible viral and cellular enhancer elements to regulate transcription of selected genes. AP-2 factors bind to the consensus sequence 5'-GCCNNNGGC-3' and activate genes involved in a large spectrum of important biological functions including proper eye, face, body wall, limb and neural tube development. They also suppress a number of genes including MCAM/MUC18, C/EBP alpha and MYC. Involved in the MTA1-mediated epigenetic regulation of ESR1 expression in breast cancer.
19	*Tfap2a*	7020		TFAP2A (Transcription Factor AP-2 Alpha) is a Protein Coding gene.Sequence-specific DNA-binding protein that interacts with inducible viral and cellular enhancer elements to regulate transcription of selected genes. AP-2 factors bind to the consensus sequence 5'-GCCNNNGGC-3' and activate genes involved in a large spectrum of important biological functions including proper eye, face, body wall, limb and neural tube development. They also suppress a number of genes including MCAM/MUC18, C/EBP alpha and MYC. AP-2-alpha is the only AP-2 protein required for early morphogenesis of the lens vesicle. Together with the CITED2 coactivator, stimulates the PITX2 P1 promoter transcription activation. Associates with chromatin to the PITX2 P1 promoter region.
	**G-protein Signaling and Receptor**			
20	*Adora2a*	135	https://doi.org/10.1038/s41398-018-0190-y	ADORA2A (Adenosine A2a Receptor) is a Protein Coding gene.Receptor for adenosine. The activity of this receptor is mediated by G proteins which activate adenylyl cyclase.
21	*Gnas*	2778	https://doi.org/10.1210/EN.2015-1326	GNAS (GNAS Complex Locus) is a Protein Coding gene.This locus has a highly complex imprinted expression pattern. It gives rise to maternally, paternally, and biallelically expressed transcripts that are derived from four alternative promoters and 5' exons. Some transcripts contain a differentially methylated region (DMR) at their 5' exons, and this DMR is commonly found in imprinted genes and correlates with transcript expression. An antisense transcript is produced from an overlapping locus on the opposite strand. One of the transcripts produced from this locus, and the antisense transcript, are paternally expressed noncoding RNAs, and may regulate imprinting in this region. In addition, one of the transcripts contains a second overlapping ORF, which encodes a structurally unrelated protein - Alex. Alternative splicing of downstream exons is also observed, which results in different forms of the stimulatory G-protein alpha subunit, a key element of the classical signal transduction pathway linking receptor-ligand interactions with the activation of adenylyl cyclase and a variety of cellular reponses.

22	*Adra1b*	147	https://doi.org/10.1096/fj.201701263RR	ADRA1B (Adrenoceptor Alpha 1B) is a Protein Coding gene.Adrenergic alpha1 receptors (alpha1-adrenoceptors) are members of the adrenergic receptor group of G-protein-coupled receptors that also includes alpha2A, alpha2B, alpha2C, beta1, beta2 and beta3. The adrenergic alpha1 receptors are further divided into three subtypes: alpha1A, alpha1B and alpha1D receptors.
23	*Rgs2*	5999	https://doi.org/10.1038/tp.2011.9	RGS4 (Regulator Of G Protein Signaling 4) is a Protein Coding gene.Inhibits signal transduction by increasing the GTPase activity of G protein alpha subunits thereby driving them into their inactive GDP-bound form. Activity on G(z)-alpha is inhibited by phosphorylation of the G-protein. Activity on G(z)-alpha and G(i)-alpha-1 is inhibited by palmitoylation of the G-protein.
24	*Ophn1*	4983	https://doi.org/10.1093/molehr/gar083	OPHN1 (Oligophrenin 1) is a Protein Coding gene.Stimulates GTP hydrolysis of members of the Rho family. Its action on RHOA activity and signaling is implicated in growth and stabilization of dendritic spines, and therefore in synaptic function. Critical for the stabilization of AMPA receptors at postsynaptic sites. Critical for the regulation of synaptic vesicle endocytosis at presynaptic terminals. Required for the localization of NR1D1 to dendrites, can suppress its repressor activity and protect it from proteasomal degradation (By similarity).
	**Cell Adhesion Signaling**			
25	*Reln*	5649	https://doi.org/10.1038/npp.2011.169	RELN (Reelin) is a Protein Coding gene.Extracellular matrix serine protease that plays a role in layering of neurons in the cerebral cortex and cerebellum. Regulates microtubule function in neurons and neuronal migration. Affects migration of sympathetic preganglionic neurons in the spinal cord, where it seems to act as a barrier to neuronal migration. Enzymatic activity is important for the modulation of cell adhesion. Binding to the extracellular domains of lipoprotein receptors VLDLR and LRP8/APOER2 induces tyrosine phosphorylation of DAB1 and modulation of TAU phosphorylation.
26	*Pcdh17*	27253	https://doi.org/10.1038/mp.2016.231	PCDH17 (Protocadherin 17) is a Protein Coding gene. Potential calcium-dependent cell-adhesion protein.
	**MAP Kinase Signaling**			
27	*Fosb*	2354	https://doi.org/10.1016/j.biopsych.2011.04.021; 10.1523/JNEUROSCI.1787-13.2014.	FOSB (FosB Proto-Oncogene, AP-1 Transcription Factor Subunit) is a Protein Coding gene.The Fos gene family consists of 4 members: FOS, FOSB, FOSL1, and FOSL2. These genes encode leucine zipper proteins that can dimerize with proteins of the JUN family, thereby forming the transcription factor complex AP-1. As such, the FOS proteins have been implicated as regulators of cell proliferation, differentiation, and transformation. Alternatively spliced transcript variants encoding different isoforms have been found for this gene. [provided by RefSeq, Jul 2008]. J Clin Endocrinol Metab. 2012 May; 97(5): E765–E770.

28	*Trib1*	10221	https://doi.org/10.1038/tp.2012.20	TRIB1 (Tribbles Pseudokinase 1) is a Protein Coding gene. dapter protein involved in protein degradation by interacting with COP1 ubiquitin ligase (PubMed:27041596). The COP1-binding motif is masked by autoinhibitory interactions with the protein kinase domain (PubMed:26455797). Serves to alter COP1 substrate specificity by directing the activity of COP1 toward CEBPA (PubMed:27041596). Binds selectively the recognition sequence of CEBPA (PubMed:26455797). Regulates myeloid cell differentiation by altering the expression of CEBPA in a COP1-dependent manner (By similarity). Controls macrophage, eosinophil and neutrophil differentiation via the COP1-binding domain (By similarity). Interacts with MAPK kinases and regulates activation of MAP kinases, but has no kinase activity (PubMed:15299019, PubMed:26455797).
29	*Sgk1*	6446	https://doi.org/10.1038/s41398-017-0026-1	SGK1 (Serum/Glucocorticoid Regulated Kinase 1) is a Protein Coding gene. This gene encodes a serine/threonine protein kinase that plays an important role in cellular stress response. This kinase activates certain potassium, sodium, and chloride channels, suggesting an involvement in the regulation of processes such as cell survival, neuronal excitability, and renal sodium excretion. High levels of expression of this gene may contribute to conditions such as hypertension and diabetic nephropathy. Several alternatively spliced transcript variants encoding different isoforms have been noted for this gene.
30	*Sik1*	150094	https://doi.org/10.1210/en.2011-1404	SIK1 (Salt Inducible Kinase 1) is a Protein Coding gene.Serine/threonine-protein kinase involved in various processes such as cell cycle regulation, gluconeogenesis and lipogenesis regulation, muscle growth and differentiation and tumor suppression. Phosphorylates HDAC4, HDAC5, PPME1, SREBF1, CRTC1/TORC1 and CRTC2/TORC2. Acts as a tumor suppressor and plays a key role in p53/TP53-dependent anoikis, a type of apoptosis triggered by cell detachment: required for phosphorylation of p53/TP53 in response to loss of adhesion and is able to suppress metastasis. Part of a sodium-sensing signaling network, probably by mediating phosphorylation of PPME1: following increases in intracellular sodium, SIK1 is activated by CaMK1 and phosphorylates PPME1 subunit of protein phosphatase 2A (PP2A), leading to dephosphorylation of sodium/potassium-transporting ATPase ATP1A1 and subsequent increase activity of ATP1A1. Acts as a regulator of muscle cells by phosphorylating and inhibiting class II histone deacetylases HDAC4 and HDAC5, leading to promote expression of MEF2 target genes in myocytes. Also required during cardiomyogenesis by regulating the exit of cardiomyoblasts from the cell cycle via down-regulation of CDKN1C/p57Kip2. Acts as a regulator of hepatic gluconeogenesis by phosphorylating and repressing the CREB-specific coactivators CRTC1/TORC1 and CRTC2/TORC2, leading to inhibit CREB activity. Also regulates hepatic lipogenesis by phosphorylating and inhibiting SREBF1. In concert with CRTC1/TORC1, regulates the light-induced entrainment of the circadian clock by attenuating PER1 induction; represses CREB-mediated transcription of PER1 by phosphorylating and deactivating CRTC1/TORC1

31	*Rbbp4*	5928	https://doi.org/10.1038/tp.2011.9	RBBP4 (RB Binding Protein 4, Chromatin Remodeling Factor) is a Protein Coding gene. Core histone-binding subunit that may target chromatin assembly factors, chromatin remodeling factors and histone deacetylases to their histone substrates in a manner that is regulated by nucleosomal DNA. Component of several complexes which regulate chromatin metabolism. These include the chromatin assembly factor 1 (CAF-1) complex, which is required for chromatin assembly following DNA replication and DNA repair; the core histone deacetylase (HDAC) complex, which promotes histone deacetylation and consequent transcriptional repression; the nucleosome remodeling and histone deacetylase complex (the NuRD complex), which promotes transcriptional repression by histone deacetylation and nucleosome remodeling; the PRC2/EED-EZH2 complex, which promotes repression of homeotic genes during development; and the NURF (nucleosome remodeling factor) complex.
**Dopamine Signaling**				
32	*Comt*	1312	https://doi.org/10.1371/journal.pone.0177506	COMT (Catechol-O-Methyltransferase) is a Protein Coding gene. Catechol-O-methyltransferase catalyzes the transfer of a methyl group from S-adenosylmethionine to catecholamines, including the neurotransmitters dopamine, epinephrine, and norepinephrine. This O-methylation results in one of the major degradative pathways of the catecholamine transmitters. In addition to its role in the metabolism of endogenous substances, COMT is important in the metabolism of catechol drugs used in the treatment of hypertension, asthma, and Parkinson disease. COMT is found in two forms in tissues, a soluble form (S-COMT) and a membrane-bound form (MB-COMT). The differences between S-COMT and MB-COMT reside within the N-termini.
33	*Dbh*	1621	https://doi.org/10.1016/j.lfs.2019.116559	DBH (Dopamine Beta-Hydroxylase) is a Protein Coding gene. Conversion of dopamine to noradrenaline.
34	*Maoa*	4128	https://doi.org/10.1093/molbev/msz061	MAOA (Monoamine Oxidase A) is a Protein Coding gene. Catalyzes the oxidative deamination of biogenic and xenobiotic amines and has important functions in the metabolism of neuroactive and vasoactive amines in the central nervous system and peripheral tissues. MAOA preferentially oxidizes biogenic amines such as 5-hydroxytryptamine (5-HT), norepinephrine and epinephrine.
35	*Maob*	4129		MAOB (Monoamine Oxidase B) is a Protein Coding gene.Catalyzes the oxidative deamination of biogenic and xenobiotic amines and has important functions in the metabolism of neuroactive and vasoactive amines in the central nervous system and peripheral tissues. MAOB preferentially degrades benzylamine and phenylethylamine.
36	*Drd1*	1812	https://doi.org/10.1038/tp.2011.9	DRD1 (Dopamine Receptor D1) is a Protein Coding gene.This gene encodes the D1 subtype of the dopamine receptor. The D1 subtype is the most abundant dopamine receptor in the central nervous system. This G-protein coupled receptor stimulates adenylyl cyclase and activates cyclic AMP-dependent protein kinases. D1 receptors regulate neuronal growth and development, mediate some behavioral responses, and modulate dopamine receptor D2-mediated events.
37	*Drd2*	1813	https://doi.org/10.1038/tp.2011.9	[illegible] subtype of the dopamine receptor. This G-protein coupled receptor inhibits adenylyl

Neuronal Growth Related Genes				
38	*Otx1*	5013	https://doi.org/10.1126/science.aan4491	OTX1 (Orthodenticle homeobox 1)The encoded protein acts as a transcription factor and may play a role in brain and sensory organ development. A similar protein in mouse is required for proper brain and sensory organ development and can cause epilepsy. Alternative splicing results in multiple transcript variants
39	*Foxp2*	93986	https://doi.org/10.1093/cercor/bhz209	FOXP2 (Forkhead box P2). The gene provides instructions for making a protein called forkhead box P2. This protein is a transcription factor, which means that it controls the activity of other genes. It attaches (binds) to the DNA of these genes through a region known as a forkhead domain. Researchers suspect that the forkhead box P2 protein may regulate hundreds of genes, although only some of its targets have been identified.The forkhead box P2 protein is active in several tissues, including the brain, both before and after birth. Studies suggest that it plays important roles in brain development, including the growth of nerve cells (neurons) and the transmission of signals between them. It is also involved in synaptic plasticity, which is the ability of connections between neurons (synapses) to change and adapt to experience over time. Synaptic plasticity is necessary for learning and memory.
40	*Fos*	2353	https://doi.org/10.1038/tp.2011.9	FOS (Fos Proto-Oncogene, AP-1 Transcription Factor Subunit) is a Protein Coding gene.The Fos gene family consists of 4 members: FOS, FOSB, FOSL1, and FOSL2. These genes encode leucine zipper proteins that can dimerize with proteins of the JUN family, thereby forming the transcription factor complex AP-1. As such, the FOS proteins have been implicated as regulators of cell proliferation, differentiation, and transformation. In some cases, expression of the FOS gene has also been associated with apoptotic cell death.
41	*Nrg1*	3084	https://doi.org/10.1096/fj.201701263RR	NRG1 (Neuregulin 1) is a Protein Coding gene.The protein encoded by this gene is a membrane glycoprotein that mediates cell-cell signaling and plays a critical role in the growth and development of multiple organ systems. An extraordinary variety of different isoforms are produced from this gene through alternative promoter usage and splicing. These isoforms are expressed in a tissue-specific manner and differ significantly in their structure, and are classified as types I, II, III, IV, V and VI. Dysregulation of this gene has been linked to diseases such as cancer, schizophrenia, and bipolar disorder (BPD).
42	*Dynll2*	140735	https://doi.org/10.1038/tp.2011.9	DYNLL2 (Dynein Light Chain LC8-Type 2) is a Protein Coding gene.Acts as one of several non-catalytic accessory components of the cytoplasmic dynein 1 complex that are thought to be involved in linking dynein to cargos and to adapter proteins that regulate dynein function. Cytoplasmic dynein 1 acts as a motor for the intracellular retrograde motility of vesicles and organelles along microtubules. May play a role in changing or maintaining the spatial distribution of cytoskeletal structures
43	*Tial1*	7073	https://doi.org/10.1530/REP-09-0373	TIAL1 (TIA1 Cytotoxic Granule Associated RNA Binding Protein Like 1) is a Protein Coding gene.RNA-binding protein. Possesses nucleolytic activity against cytotoxic lymphocyte target cells. May be involved in apoptosis.

44	*Fabp5*	2171	https://doi.org/10.1016/j.neures.2014.08.012.	FABP5 (Fatty Acid Binding Protein 5) is a Protein Coding gene.This gene encodes the fatty acid binding protein found in epidermal cells, and was first identified as being upregulated in psoriasis tissue. Fatty acid binding proteins are a family of small, highly conserved, cytoplasmic proteins that bind long-chain fatty acids and other hydrophobic ligands. FABPs may play roles in fatty acid uptake, transport, and metabolism. Polymorphisms in this gene are associated with type 2 diabetes. The human genome contains many pseudogenes similar to this locus.[provided by RefSeq, Feb 2011]
45	*Rnf141*	50862	https://doi.org/10.1016/j.modgep.2007.10.007	RNF141 (Ring Finger Protein 141) is a Protein Coding gene.May be involved in spermatogenesis.
	**Housekeeping genes**			
46	*Gapdh*	2597		GAPDH (Glyceraldehyde-3-Phosphate Dehydrogenase) is a Protein Coding gene.
47	*Tbp*	6908		TBP (TATA-Box Binding Protein) is a Protein Coding gene.
48	*Ppia*	5478		PPIA (Peptidylprolyl Isomerase A) is a Protein Coding gene.

Green = Transgenerational (CD+Veh vs CD+DHT) (Risal S. et al ., 2019)
Yellow= CD+Veh vs HFHS+Veh (Risal S. et al ., 2019)
Blue= Expressed in serum of PCOS-D (Risal S. et al ., 2019)
Non-highlighted= Publication (please see DOI)
